# Supplementary material for: Leveraging Nutrient-Rich Traditional Foods to Improve Diets among Indigenous Populations in India: Value Chain Analysis of Finger Millet and Kionaar Leaves
Source: Foods. 2022 Nov 23;11(23):3774. doi: 10.3390/foods11233774 (PMC9736962; doi:10.3390/foods11233774)
Supplement: Supplementary file 1 [file foods-11-03774-s001.zip › foods-1945887-supplementary.pdf]

Supplementary Table S1. Demographic information for interview participants.

Tribal group	Participant ID	Block	Village	Age	Gender	Type of Respondent
Munda	2001	Murhu	Charid	35	Female	Health and nutrition worker
Munda	2002	Murhu	Charid	55	Male	Village head
Munda	2003	Murhu	Kudapurti	32	Female	Health and nutrition worker
Munda	2004	Murhu	Kudapurti	47	Male	Village head
Munda	2011	Murhu	Charid	55	Male	Village head
Munda	2012	Murhu	Kudapurti	60	Male	Elder village man
Munda	2005	Torpa	Tati	52	Female	Health and nutrition worker
Munda	2006	Torpa	Tati	55	Male	Village head
Munda	2007	Torpa	Tati	54	Male	Village head
Munda	2008	Torpa	Nichitpur	45	Female	Health and nutrition worker
Munda	2009	Torpa	Nichitpur	35	Female	Village woman
Munda	2010	Torpa	Nichitpur	60	Male	Village head
Sauria Paharia	1001	Sunderpahari	Chewo	36	Female	Health and nutrition worker
Sauria Paharia	1002	Sunderpahari	Chewo	24	Female	Village woman
Sauria Paharia	1003	Sunderpahari	Chewo	27	Female	Village Head
Sauria Paharia	1004	Sunderpahari	Chewo	21	Female	Village woman
Sauria Paharia	1005	Sunderpahari	Chewo	65	Female	Elderly village woman
Sauria Paharia	2001	Sunderpahari	Chota Haripur	56	Female	Elderly village woman
Sauria Paharia	2002	Sunderpahari	Chota Haripur	27	Female	Health and nutrition worker
Sauria Paharia	2003	Sunderpahari	Chota Haripur	24	Female	Village woman
Sauria Paharia	2004	Sunderpahari	Chota Haripur	38	Female	Village woman
Sauria Paharia	2005	Sunderpahari	Chota Haripur	35	Female	Village woman
Sauria Paharia	3001	Boarijor	Kusumghati	35	Female	Village woman
Sauria Paharia	3002	Boarijor	Kusumghati	34	Female	Health and nutrition worker
Sauria Paharia	3003	Boarijor	Kusumghati	60	Female	Elderly village woman
Sauria Paharia	3004	Boarijor	Kusumghati	32	Female	Village woman
Sauria Paharia	3005	Boarijor	Kusumghati	30	Female	Village woman
Sauria Paharia	4001	Boarijor	Teletok	60	Female	Elderly village woman
Sauria Paharia	4002	Boarijor	Teletok	28	Female	Health and nutrition worker
Sauria Paharia	4003	Boarijor	Teletok	24	Female	Village woman
Sauria Paharia	4004	Boarijor	Teletok	32	Female	Village woman
